# Structural diversity across arbuscular mycorrhizal, ectomycorrhizal, and endophytic plant–fungus networks

**DOI:** 10.1186/s12870-018-1500-5

**Published:** 2018-11-21

**Authors:** Hirokazu Toju, Hirotoshi Sato, Satoshi Yamamoto, Akifumi S. Tanabe

**Affiliations:** 1 Center for Ecological Research, Kyoto University, Otsu, Shiga 520-2113 Japan; 2 Precursory Research for Embryonic Science and Technology (PRESTO), Japan Science and Technology Agency, Kawaguchi, Saitama 332-0012 Japan; 3 Graduate School of Human and Environmental Studies, Kyoto University, Sakyo, Kyoto 606-8501 Japan; 4 Graduate School of Science, Kyoto University, Kitashirakawa-oiwake-cho, Kyoto, 606-8502 Japan; 5 Faculty of Science and Technology, Ryukoku University, 1-5 Yokotani, Seta Oe-cho, Otsu, Shiga 520-2194 Japan

**Keywords:** Biodiversity, Community ecology, Competitive exclusion, Host specificity or preference, Latitudinal gradients, Microbiomes, Plant–fungus interactions, Plant–soil feedback, Species coexistence, Mycorrhizal and endophytic symbiosis

## Abstract

**Background:**

Below-ground linkage between plant and fungal communities is one of the major drivers of terrestrial ecosystem dynamics. However, we still have limited knowledge of how such plant–fungus associations vary in their community-scale properties depending on fungal functional groups and geographic locations.

**Methods:**

By compiling a high-throughput sequencing dataset of root-associated fungi in eight forests along the Japanese Archipelago, we performed a comparative analysis of arbuscular mycorrhizal, ectomycorrhizal, and saprotrophic/endophytic associations across a latitudinal gradient from cool-temperate to subtropical regions.

**Results:**

In most of the plant–fungus networks analyzed, host–symbiont associations were significantly specialized but lacked “nested” architecture, which has been commonly reported in plant–pollinator and plant–seed disperser networks. In particular, the entire networks involving all functional groups of plants and fungi and partial networks consisting of ectomycorrhizal plant and fungal species/taxa displayed “anti-nested” architecture (i.e., negative nestedness scores) in many of the forests examined. Our data also suggested that geographic factors affected the organization of plant–fungus network structure. For example, the southernmost subtropical site analyzed in this study displayed lower network-level specificity of host–symbiont associations and higher (but still low) nestedness than northern localities.

**Conclusions:**

Our comparative analyses suggest that arbuscular mycorrhizal, ectomycorrhizal, and saprotrophic/endophytic plant–fungus associations often lack nested network architecture, while those associations can vary, to some extent, in their community-scale properties along a latitudinal gradient. Overall, this study provides a basis for future studies that will examine how different types of plant–fungus associations collectively structure terrestrial ecosystems.

**Electronic supplementary material:**

The online version of this article (10.1186/s12870-018-1500-5) contains supplementary material, which is available to authorized users.

## Background

Fungi in the below-ground biosphere are key drivers of terrestrial ecosystem processes [1–4]. Mycorrhizal fungi are considered to support land plants not only by provisioning soil nitrogen and phosphorous [5, 6] but also by increasing plants’ resistance to biotic/abiotic stress [7, 8]. Pathogenic fungi in the soil affect the survival/mortality of young plants in a major way, possibly determining spatial distributions of plant species within forest/grassland ecosystems [9, 10]. Moreover, recent mycological studies have begun to examine the poorly explored diversity of endophytic fungi, which can enhance the nutritional conditions and pathogen resistance of mycorrhizal and non-mycorrhizal plant species [11–16]. Thus, terrestrial biomes consist of multiple layers of below-ground plant–fungus interactions [17]. Nonetheless, we still have limited knowledge of the structure of such complex webs of interactions, leaving major processes in below-ground ecosystems poorly explored.

In enhancing our understanding of community- or ecosystem-level processes of below-ground plant–fungus interactions, analyses on community-scale properties of such host–symbiont associations provide essential insights. For example, if a pathogenic fungal community consists mainly of species with narrow host ranges, it as a whole is expected to restrict the emergence of dominant plant species through “negative plant–soil feedback”, contributing to the maintenance of plant species diversity within an ecosystem [18–20]. In contrast, with a high proportion of mycorrhizal fungi with narrow host ranges, their specific host species, such as Pinaceae plants hosting Suillaceae ectomycorrhizal fungi [21], will dominate the plant community through positive plant–soil feedback [20, 22]. Meanwhile, endophytic and arbuscular mycorrhizal fungi with broad host ranges [23–25] may diminish such negative and positive feedback by interlinking otherwise compartmentalized ecological dynamics (but see [26]). Therefore, concomitant analyses of community-scale properties of those multiple plant–fungus associations are of particular importance in understanding how plant–soil feedbacks organize terrestrial ecological processes.

Since the application of network science to ecology and mycology, researchers have evaluated the architecture of networks that represent linkage between plant and fungal communities [27]. Those studies have shown that arbuscular mycorrhizal [28–30], ectomycorrhizal [31], and ericaceous [32] plant–fungus networks exhibit moderate or low levels of host–symbiont specificity, while they are structured to avoid overlap of host plant ranges within fungal communities. In addition, many of those plant–fungus networks [17, 31, 33] are known to lack “nested” architecture (i.e., structure of networks wherein specialist species interact with subsets of partners of generalist species [34]), which has been commonly reported in above-ground networks of plant–pollinator and plant–seed-disperser interactions [34–36] (but see [37]). However, in those previous studies, data of different types of plant–fungus networks have been collected from different geographic localities with different sampling strategies, precluding the chance of simultaneously evaluating the effects of interaction type and geographic factors. Although comparative studies of published data provide invaluable insights [27], compiled data often vary in the molecular markers used and they may differ in appropriate null model assumptions in statistically examining network topological properties.

In this study, we compared community-scale properties of arbuscular-mycorrhizal, ectomycorrhizal, and endophytic associations across eight forest sites spanning from cool-temperate to subtropical regions in Japan. Based on high-throughput sequencing data of root-associated fungi [38], we analyzed how multiple plant species are associated with respective functional groups of fungi in each of the eight forests. We then examined how network structure varied depending on categories of plant–fungus associations and geographic locations. Overall, this study provides a first step for integrating insights into community-scale properties of multiple types of below-ground plant–fungus associations and their ecosystem-level consequences.

## Methods

### Terminology

In analyzing metadata of community-scale properties of plant–fungus associations, we need to use consistent terminology that can be applied to a wide range of host–symbiont associations. While plant–fungus network properties have been compared within a single functional group of fungi (e.g., arbuscular mycorrhizal or ectomycorrhizal fungi) in most studies, we herein target not only arbuscular mycorrhizal and ectomycorrhizal fungi but also pathogenic and saprotrophic/endophytic fungi. The dataset used in this study [38] included all the fungi detected by high-throughput sequencing and they could contain not only mutualistic/antagonistic fungi but also commensalistic fungi merely adhering to plant roots [39]. In this sense, our data represented symbiotic relationships in the broad sense, i.e., intimate physical connections between organisms [17, 40].

### Sampling

**Fig. 1:**
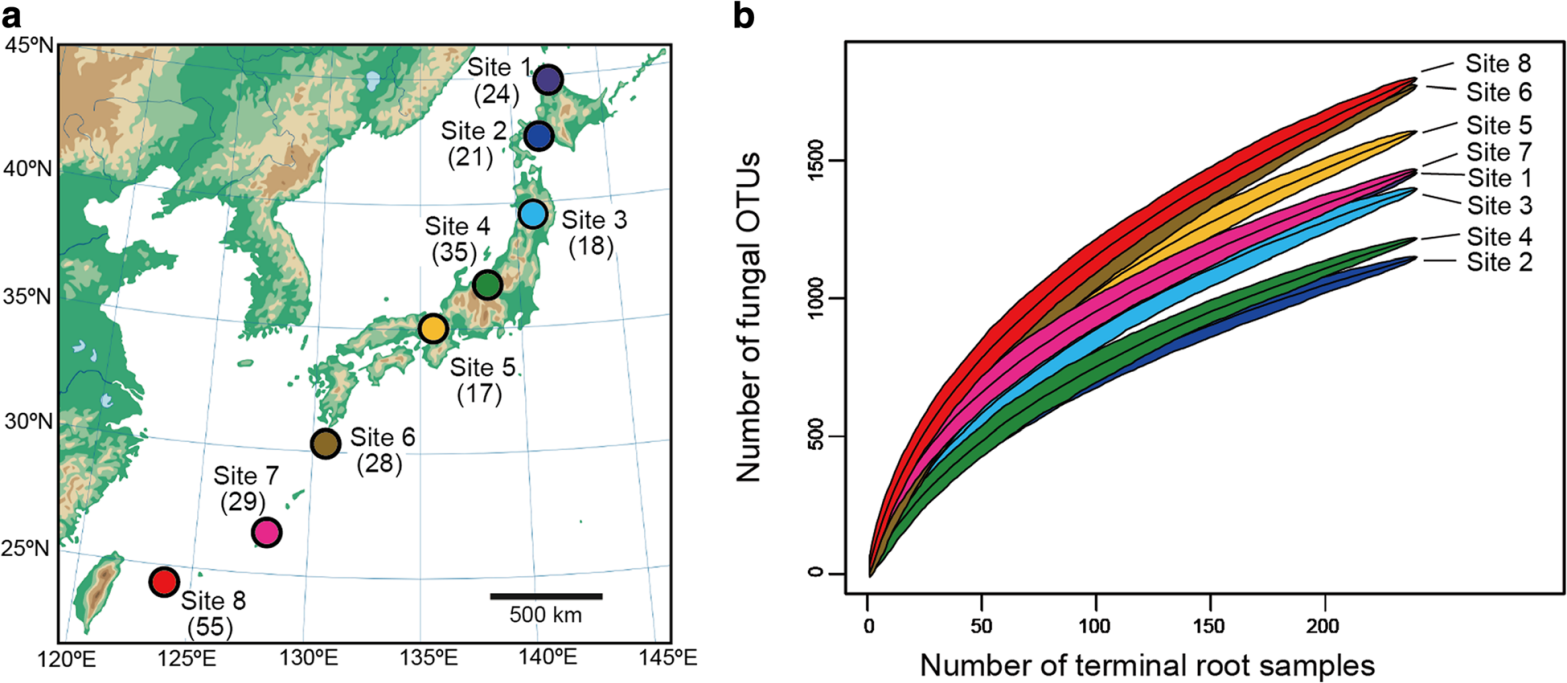
Study sites. **a** Map of study sites. In each forest site, a number in a parenthesis indicates the number of plant species/taxa observed in the 240 terminal root samples from which sequencing data were successfully obtained. The map published by DesignEXchange Co., Ltd. was purchased by the corresponding author (H.T.), who has the right to edit and publish it. **b** Relationship between the number of samples and that of plant species/taxa observed. A rarefaction curve obtained from 240 terminal-root samples is shown for each study site

We used the dataset of a previous study [38], in which we collected root samples at eight forest sites (four cool-temperate, one warm-temperate, and three subtropical forests) across the entire range of the Japanese Archipelago (45.042–24.407 °N; Fig. 1a; Additional file 1: Data S1) in order to infer metacommunity processes of plant–fungus associations. In each forest, 2-cm segment of terminal roots were collected from 3-cm below the soil surface at 1-m horizontal intervals: 383 terminal root samples were collected in each of the eight forests. Those roots were collected indiscriminately regarding root morphology or apparent mycorrhizal type so that the samples as a whole represented the relative frequency of plant–fungal associations in the horizon in each forest [41]. Therefore, while the sample sets consisted mainly of woody plants, they also included herbaceous plants (Additional file 2: Data S2). Each root sample was preserved in 70% ethanol and stored at − 25 °C until DNA extraction. Research permits were issued by the organizations listed in Acknowledgements.

### Molecular and bioinformatic analyses

The molecular and bioinformatic analyses were performed as detailed below and in the data source study [38]. Each root sample was placed in 70% ethanol with 1-mm zirconium balls in a 1.5 mL tube. The 1.5 mL tubes were then shaken at 15 Hz for 2 min with a TissueLyser II (Qiagen) [23]. The washed roots were subsequently pulverized by shaking with 4-mm zirconium balls at 25 Hz for 3 min. DNA extraction was then performed with a cetyltrimethylammonium bromide method [42].

The internal transcribed spacer 1 (ITS1) region of root-associated fungi was amplified with the primers ITS1F_KYO1 and ITS2_KYO2, which target not only Ascomycota and Basidiomycota fungi but also diverse non-Dikarya (e.g., Glomeromycota) taxa [43]. We used the forward primer ITS1F_KYO1 fused with 3–6-mer Ns for improved Illumina sequencing quality [44] and the forward Illumina sequencing primer (5′- TCG TCG GCA GCG TCA GAT GTG TAT AAG AGA CAG- [3–6-mer Ns] – [ITS1F_KYO1] -3′) and the reverse primer ITS2_KYO2 fused with 3–6-mer Ns and the reverse sequencing primer (5′- GTC TCG TGG GCT CGG AGA TGT GTA TAA GAG ACA G [3–6-mer Ns] - [ITS2_KYO2] -3′). The DNA polymerase system of KOD FX Neo (Toyobo) was used with a temperature profile of 94 °C for 2 min, followed by 35 cycles at 98 °C for 10 s, 50 °C for 30 s, 68 °C for 50 s, and a final extension at 68 °C for 5 min. The ramp rate was set to 1 °C/sec to prevent the generation of chimeric sequences [45]. Illumina sequencing adaptors were then added to each sample in the subsequent PCR using the forward primers consisting of the P5 Illumina adaptor, 8-mer tags for sample identification [46], and a partial sequence of the sequencing primer (5′- AAT GAT ACG GCG ACC ACC GAG ATC TAC AC - [8-mer index] - TCG TCG GCA GCG TC -3′) and the reverse primers consisting of the P7 adaptor, 8-mer tags, and a partial sequence of the sequencing primer (5′- CAA GCA GAA GAC GGC ATA CGA GAT - [8-mer index] - GTC TCG TGG GCT CGG -3′). In the reaction, KOD FX Neo was used with a temperature profile of 94 °C for 2 min, followed by 8 cycles at 98 °C for 10 s, 55 °C for 30 s, 68 °C for 50 s, and a final extension at 68 °C for 5 min. The PCR amplicons of 384 samples in each forest (including one PCR negative control) were pooled with equal volume after a purification/equalization process with AMPureXP Kit (Beckman Coulter).

For the identification of plants, another set of PCR was performed targeting chloroplast *rbcL* region with rbcL_F3 and rbcL_R4 primers [41]. The fusion primer design, DNA polymerase system, temperature profiles, and purification processes used in the *rbcL* analysis were the same as those of the fungal ITS analysis. The ITS and *rbcL* libraries were processed in two Illumina MiSeq runs, in each of which samples of four forest sites were combined (run center: KYOTO-HE) (2 × 250 cycles; 15% PhiX spike-in).

In total, 17,724,456 and 17,228,848 reads were obtained for the first and second MiSeq runs. The raw sequencing data were converted into FASTQ files using the program bcl2fastq 1.8.4 provided by Illumina. The FASTQ files were then demultiplexed using the program Claident v0.2.2016.07.05 [47, 48]. To avoid possible errors resulting from low-quality index sequences, the sequencing reads whose 8-mer index positions included nucleotides with low (< 30) quality scores were discarded in this process. As reverse sequences output by Illumina sequencers have lower quality values than forward sequences [49], we used only forward sequences after removing low-quality 3′-ends using Claident (sequencing data deposit: DDBJ DRA accession: DRA006339) [38]. Noisy reads were subsequently discarded and the reads that passed the filtering process were clustered using VSEARCH [50] as implemented in Claident. The threshold sequencing similarities in the clustering were set to 97% for fungal ITS and 98% for *rbcL*, respectively. While sequence similarity values have been set to 97% in most ITS analyses of Ascomycota and Basidiomycota fungi [51] (see also [52]), a recent study showed that Glomeromycota fungi generally had much higher intraspecific ITS-sequence variation than Dikarya fungi [53]. Therefore, we performed an additional clustering analysis with a 94% cutoff similarity for defining Glomeromycota OTUs. Note that changing cut-off similarities (81–97%) did not qualitatively change statistical properties of a plant–fungus network in a previous study [17]. The taxonomic assignment of the OTUs (Additional files 3 and 4: Data S3–4) was conducted based on the combination of the query-centric auto-*k*-nearest neighbor (QCauto) method [47] and the lowest common ancestor (LCA) algorithm [54] as implemented in Claident. Note that taxonomic identification results based on the QCauto–LCA approach were comparable to, or sometimes more accurate than, those with the alternative approach combining the UCLUST algorithm [55] with the UNITE database [56] [see [32, 57] for detailed comparison of the QCautoLCA and UCLUST–UNITE approaches]. The functional group of each fungal OTU was inferred using the program FUNGuild 1.0 [58]. For 44.1% (3560/8080) of fungal OTUs, functional group information was inferred (Additional file 1: Data S1).

The obtained information of *rbcL* OTUs was used to identify each root sample, although species-level taxonomic information was unavailable for some plant taxa in each forest due to the relatively low variability of the chloroplast region [59]. Thus, we also used the information of the ITS sequencing libraries, which included not only fungal but also host plant sequencing reads: there were plant taxa that could not be identified to species even with ITS information. Based on the *rbcL* and ITS information of plant sequences, possibly contaminated samples were removed from the dataset.

For each of the eight forests, we then obtained a sample (row) × fungal OTU (column) data matrix, in which a cell entry depicted the number of sequencing reads of an OTU in a sample. The cell entries whose read counts represented less than 0.1% of the total read count of each sample were subsequently excluded because those rare entries could derive from contaminations from soil or PCR/sequencing errors [60]. The filtered matrices were then rarefied to 1000 reads per sample using the “rrarefy” function of the vegan 2.4–3 package [61] of R 3.4.1 [62]. As the number of samples with 1000 or more reads varied among the eight forests examined (240–288 samples), it was equalized by randomly sampling 240 samples without duplication in each forest (“sample-level matrices”; Additional file 2: Data S2).

Based on the sample-level matrix of each forest, we obtained an additional matrix, in which a cell indicated the number of samples representing associations between a plant species/taxa (row) and a fungal OTU (column) (“species-level matrices”; Additional file 5: Data S5). In addition to the matrix indicating associations between all fungal OTUs and their host plants (ALL), a series of partial network matrices representing respective fungal functional groups were obtained by selecting arbuscular mycorrhizal (AM), ectomycorrhizal (ECM), potentially pathogenic (PATHO), and saprotrophic/endophytic (SAPENDO) fungal OTUs in each forest (Additional file 6: Data S6). Due to the limited availability of information of fungal ecology, functional groups of many fungal OTUs could not be estimated and there were only 9–25 fungal OTUs inferred to be plant pathogens in respective forests (Additional files 1 and 5: Data S1 and S5). For arbuscular mycorrhizal symbiosis, we prepared additional matrices from which non-arbuscular mycorrhizal plants [63] were excluded (AM.ex partial networks). Likewise, for ectomycorrhizal symbiosis, we obtained additional matrices from which non-ectomycorrhizal plants were excluded (EcM.ex partial networks): a list of ectomycorrhizal plants [63] was referred to in classifying ectomycorrhizal and non-ectomycorrhizal plants. Although some plant species are known to interact with both arbuscular mycorrhizal and ectomycorrhizal fungi [64], matrices consisting exclusively of arbuscular mycorrhizal plants and fungi (AM.ex) and those consisting exclusively of ectomycorrhizal plants and fungi (EcM.ex) (Additional file 6) likely represented what generally regarded as arbuscular mycorrhizal or ectomycorrhizal symbioses.

### Data analysis

Based on the sample-level matrices, relationships between the number of samples and that of observed fungal OTUs was analyzed for each forest using the “specaccum” function of the vegan package. The community-scale plant–fungus associations represented by the species-level matrices (“ALL” network matrices; Additional file 5: Data S5) were visualized using the program GePhi 0.9.1 [65] with “ForceAtlas2” layout algorithm [66]. We then analyzed the statistical properties of the ALL networks and partial networks (Additional file 6: Data S6) in terms of the metric of network-level interaction specificity (*H*_*2*_*’*) [67], which has been frequently used to measure the degree of interaction specificity in host–symbiont networks [68, 69]. The plant–fungus associations were evaluated also by the weighted NODF metric [70] of network nestedness [34], which measures the degree to which specialists (species with narrow partner ranges) interact with partners of generalists (species with broad partner ranges) in the same guild or trophic level. We further examined how host plant ranges were differentiated within the fungal community of each forest based on checkerboard scores [71]: a high/low score of the checkerboard index indicates host differentiation/overlap within a guild or trophic level [69]. Although modularity is another important index frequently used in ecological network studies [35], its computation was too time-consuming to be applied to randomization analyses (see below) of our present data consisting of more than 1000 fungal OTUs and their host plants. Note that we previously found that below-ground plant–fungal associations generally showed statistically significant but low network modularity [17, 32, 69].

As estimates of network indices could vary depending on species compositions of examined communities, we standardized the indices as

$$ \mathrm{relative}\ \mathrm{index}\ \mathrm{value}=\left[{I}_{observed}-\mathrm{mean}\left({I}_{randomized}\right)\right]/\mathrm{SD}\left({I}_{randomized}\right), $$

where *I*_observed_ was the index estimate of the observed data matrix, and mean(*I*_randomized_) and SD(*I*_randomized_) were the mean and standardized deviation of the index values of randomized matrices [69]. Randomized matrices were obtained by shuffling host-plant labels in the sample-level matrices and subsequently converting the randomized sample-level matrices into randomized species-level matrices. Although we used two additional methods [“r2dtable” [72] and “vaznull” [73] methods] of matrix randomization in our previous studies of plant–fungus networks [17, 69], they were too time-consuming to be used in the present large dataset: note that the three randomization methods compared in those previous studies yielded qualitatively similar results [17, 69]. The number of randomizations was set to 1000 for *H*_*2*_*’*/nestedness analyses and 100 for checkerboard-score analyses, which required substantial computing time.

Based on the network indices, we examined how the community-scale properties of the plant–fungus associations varied among local forests and network categories (ALL, AM, AM.ex, EcM, EcM.ex, SAPENDO, and PATHO). For interaction specificity (relative *H*_*2*_*’*), nestedness (relative weighted NODF nestedness), and checkerboard index (relative checkerboard values) each, an ANOVA model was constructed by incorporating locality (forest sites), network category, number of plant species/taxa, number of fungal OTUs, and network connectance (the proportion of non-zero entries in community matrices) as explanatory variables. The variation in the plant–fungus network properties was visualized based on a principal component analysis based on a correlation matrix: the variables included were *H*_*2*_*’* interaction specificity, NODF nestedness, checkerboard index, number of plant species/taxa, number of fungal OTUs, proportion of fungal OTUs to plant species/taxa, and connectance.

## Results

**Fig. 2:**
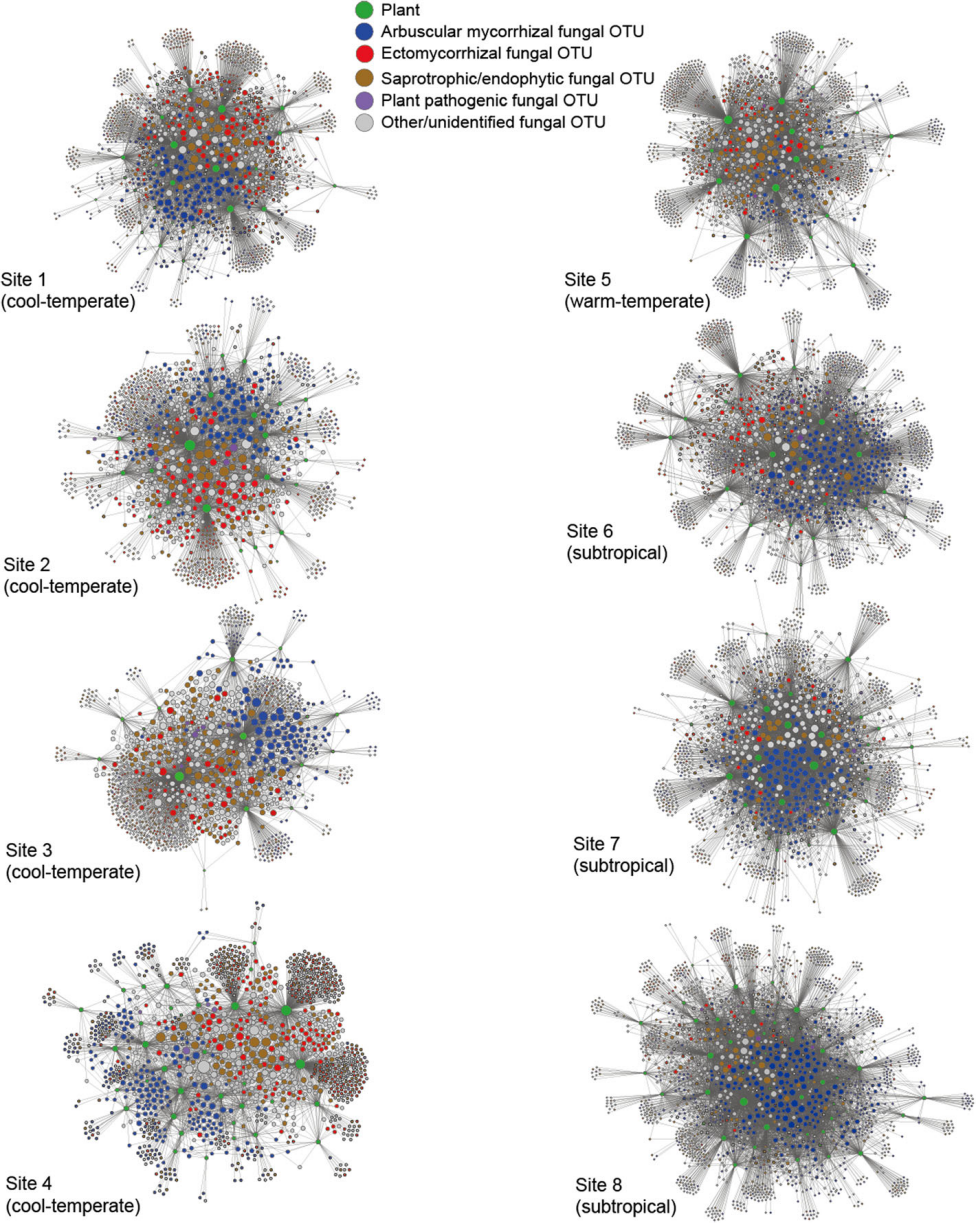
Below-ground plant–fungus networks. The “ALL” network involving all the root-associated fungal OTUs detected and their host plant species/taxa is shown for each forest. The OTUs/species in the networks are arranged with the “ForceAtlas2” layout algorithm [66]. Size of circles represents betweenness centrality scores compared within plant/fungal community

**Fig. 3:**
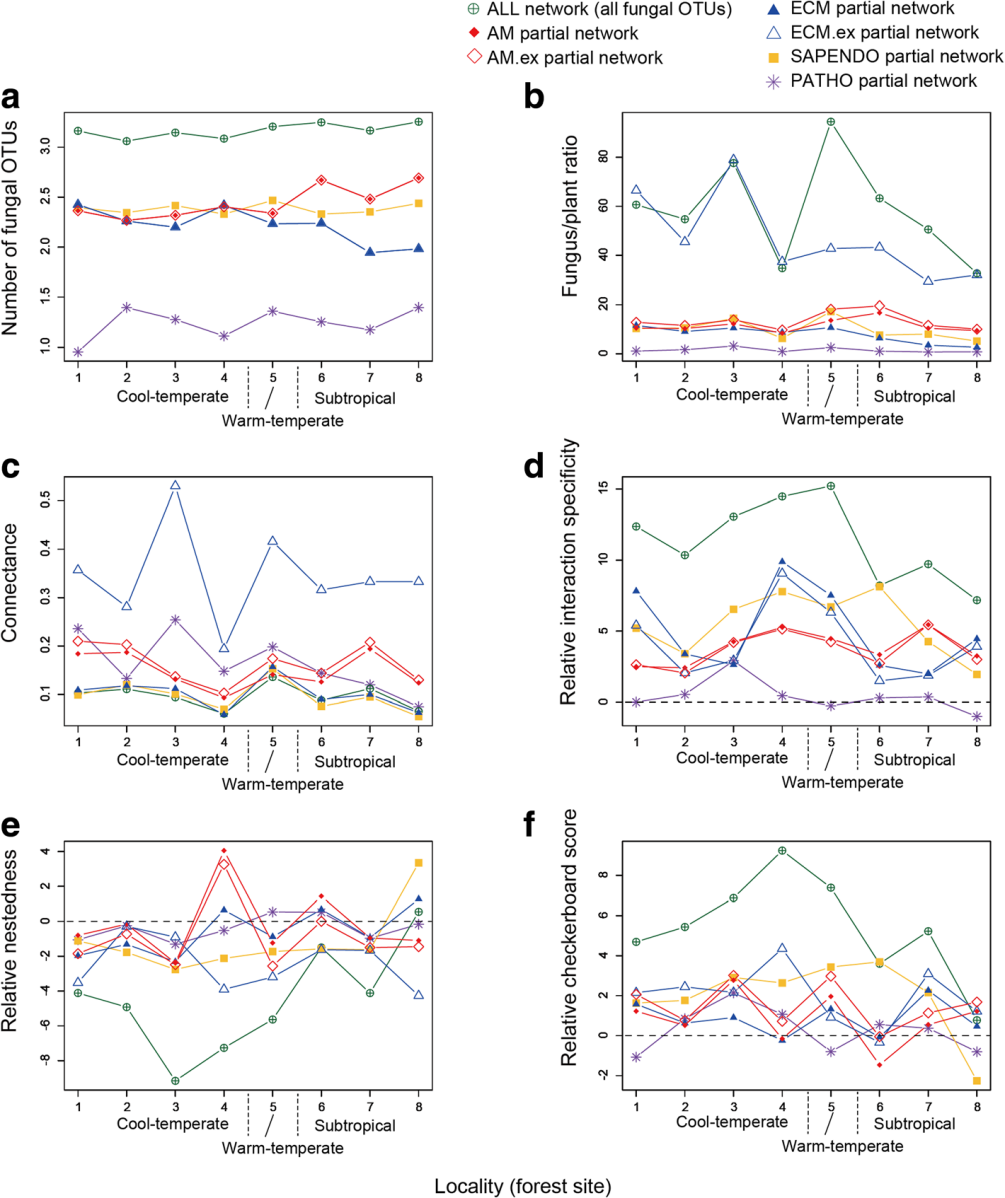
Network properties. The index scores representing the architecture of plant fungus networks/partial networks are shown across the eight forests examined. **a** The number of fungal OTUs. The code numbers of forest sites correspond to those shown in Fig. 1. **b** The ratio of the number of fungal OTUs to that of the plant species/taxa involved in each network/partial network. **c** Connectance (the proportion of non-zero entries in a species-level matrix). **d** Network-level interaction specificity (relative *H*_*2*_*’*). **e** Nestedness (relative weighted NODF nestedness). **f** Host range differentiation (relative checkerboard score). For relative interaction specificity, relative nestedness, and relative checkerboard score (**d**-**f**), scores higher/lower than 2 roughly indicate that observed network index values are higher/lower than expected by chance (see Additional file 7: Data S7 for detailed results of the randomization test)

Total fungal OTU richness was higher in warm-temperate and subtropical forests than in cool-temperate forests (Figs. 1b and 2). The OTU richness of arbuscular mycorrhizal fungi was higher in the three subtropical forests, while that of ectomycorrhizal fungi decreased in the subtropical forests (Fig. 3a). The ratio of the total number of fungal OTUs to the number of plant species/taxa varied, to some extent, among forests, although there was seemingly no systematic variation between cool-temperate and the other (warm temperate and subtropical) localities (Fig. 3b). Connectance varied among forests as well, while it was consistently higher in EcM.ex partial networks than in other networks/partial networks (Fig. 3c). We also found that AM and AM.ex partial networks showed higher connectance than ALL, EcM, and SAPENDO networks/partial networks in seven of the eight forests examined (Fig. 3c). The connectance of PATHO partial networks varied considerably among forests presumably due to low OTU richness and the resultant uncertainty in index estimation.

The relative interaction specificity significantly varied among forests and network categories in an ANOVA model (Table 1; Fig. 3d). The relative nestedness of the ALL matrices of plant–fungus associations was lower than zero in most forests but not in the southern most subtropical forest (Fig. 3e; Additional file 7: Data S7). Overall, plant–fungus associations in ALL networks were more specialized (Fig. 3d) and less nested (Fig. 3e) than those of partial networks. In addition, fungal OTUs in ALL networks displayed stronger differentiation of host ranges than those in partial networks (Fig. 3f).

**Table 1:** Potential factors contributing to variation in plant–fungus network structure. For each response variable representing network structure, an ANOVA model including the number of plant species/taxa, that of fungal OTUs, network connectance, sampling locality, and the category of plant–fungus networks was constructed

Response variable	Explanatory variable	df	*F*	*P*
Relative interaction specificity	No. plant species/taxa	1	6.8	0.0126
	No. fungal OTUs	1	120.9	**< 0.0001**
	Connectance	1	5.5	0.0238
	Locality	7	4.6	**0.0008**
	Category	6	5.0	**0.0007**
Relative nestedness	No. plant species/taxa	1	7.7	**0.0083**
	No. fungal OTUs	1	37.0	**< 0.0001**
	Connectance	1	0.5	0.4829
	Locality	7	1.3	0.2957
	Category	6	1.5	0.2042
Relative checkerboard score	No. plant species/taxa	1	1.3	0.2594
	No. fungal OTUs	1	68.8	**< 0.0001**
	Connectance	1	4.1	0.0506
	Locality	7	2.0	0.0818
	Category	6	3.1	0.014

*P* values significant after a Bonferroni correction are shown in bold for each ANOVA model (*α* = 0.05)

After taking into account plant and fungal diversity in an ANOVA model, neither locality nor network category explained the variation in relative nestedness (Table 1). The relative checkerboard scores varied among localities (Fig. 3f), although the effects of locality were non-significant in an ANOVA model (Table 1). The ANOVA model showed that the variation in relative checkerboard scores was explained, to some extent, by network category: the effects of network categories were non-significant after a Bonferroni correction (Table 1).

In the principal component analysis of network indices, ALL, PATHO and other networks/partial networks were separated by the first principal component, which represented high fungal OTU richness, fungus/plant ratios, relative interaction specificity, and relative checkerboard scores as well as low relative nestedness (Additional file 8: Figure S1a). In addition, EcM.ex partial networks were separated from other networks/partial networks by the second principal component, which represented high numbers of plant species/taxa and low connectance (Additional file 8: Figure S1a). The third principal component (Additional file 8: Figure S1b), which were negatively correlated with fungal OTU richness, fungus/plant ratios, and relative nestedness, added little to the results of grouping based on the first and second principal components.

## Discussion

The data compiled in this study [38], which included 17–55 plant species/taxa and more than 1000 fungal OTUs in each of the eight forests, provided a novel opportunity to evaluate how different types of below-ground plant–fungus associations varied in their community-scale characteristics along a latitudinal gradient. We then found that network structural properties differed among different types of plant–fungus associations (Fig. 3), while geographic factors contributed to the variation found in network structure (Table 1). Specifically, ectomycorrhizal partial networks defined in terms of both plant and fungal functional groups (EcM.ex) had higher connectance than other networks/partial networks (Additional file 8: Figure S1). We also found that networks consisting of all functional groups of fungi and their host plants had higher network-level interaction specificity, more differentiated host ranges between fungi, and lower network nestedness than the partial networks of arbuscular mycorrhizal, ectomycorrhizal, and saprotrophic/endophytic associations (Fig. 3; Additional file 8: Figure S1). As in previous studies, our data included many fungal OTUs unassigned to functional groups due to the paucity of the information on fungal functions and guilds in databases [58]. However, by extending findings in previous plant–fungus network studies [30, 31, 69], in which sampling strategies, interaction type, or geographic factors were not controlled systematically, this study offers a basis for discussing how different types of below-ground plant–fungus associations collectively build plant–soil feedbacks in terrestrial ecosystems.

Among the network indices examined in this study, nestedness showed an idiosyncratic tendency in light of other types of interaction networks examined in community ecology [34–36]. We found that below-ground plant–fungus networks often displayed “anti-nested” architecture, in which scores representing nested network structure were lower than those expected by chance (i.e., negative values of relative nestedness; Fig. 3e), as suggested also in previous studies [17, 31, 69]. In particular, the entire networks involving all plants and fungi and ectomycorrhizal partial networks defined in terms of both plant and fungal functional groups (EcM.ex) had strong anti-nested architecture in many of the forests examined (Fig. 3e). Although factors organizing anti-nested network architecture remain to be investigated, competition for host plants among fungal species has been inferred to decrease nestedness of plant–fungus associations [69]. In addition, a previous comparative study suggested that plant–fungus network nestedness decreased with increasing annual mean temperature on a global scale [27].

The prevalence of anti-nested or non-nested network structures in below-ground plant–fungus associations is in sharp contrast to observations on other types of plant–partner networks, which commonly show statistically significant nested architecture [34]. Specifically, plant–pollinator and plant–seed disperser interactions are generally characterized by nested network architecture in which overlap of partner ranges within the same guild are expected to mitigate competition between plant species [34–36]. In this sense, the anti-nested structure found in plant–fungus networks highlights potential diversity of network architecture and mechanisms by which species coexistence is promoted in plant–partner networks [36, 37, 74]. Given that below-ground fungi constitute one of the most species-rich components of the terrestrial biosphere [3], understanding community-scale properties of below-ground plant–fungus associations is a major step for disentangling relationship among network structure, species coexistence, and community stability.

To overcome the inconsistency between theory and observations, we may need to take into account basic biology of below-ground plant–fungus associations. We here highlight two backgrounds that need more attention for deepening discussion on ecological networks and species coexistence. First, in contrast to plant–pollinator or plant–seed disperser networks, which are often assumed to consist only of mutualistic interactions, below-ground plant–fungus networks can involve not only mutualistic but also antagonistic and commensalistic interactions. Even within a network consisting exclusively of arbuscular mycorrhizal or ectomycorrhizal plant and fungal species (e.g., AM.ex or EcM.ex partial networks in this study), plant–fungus interactions can have not only positive (mutualistic) but also net negative/neutral effects [75–77]. This diversity of interaction type can lead to high stability of below-ground fungal and host plant communities. Specifically, while communities consisting exclusively of mutualistic interactions are inherently unstable [78], involvement of a small fraction of antagonistic interactions in those communities can dramatically enhance species coexistence [79]. Second, because fungi can disperse long distances as spores [80, 81] (but see [82]), their local species richness (alpha diversity) may be greatly impacted by metacommunity processes [75]. Interestingly, a recent theoretical study on food webs predicted that strong coupling of local communities within a metacommunity could result in positive relationship between species richness and community stability [83]. Such theoretical evaluation of metacommunity dynamics has been extended to systems involving mutualistic interactions [84], providing platforms for considering how dispersal abilities of constituent species determine local species richness/coexistence of different types of plant–partner networks.

The dataset compiled in this study included plant–fungus combinations that could not be classified into well-recognized categories of mycorrhizal symbioses [8]. For example, ectomycorrhizal fungi were detected not only from plant species in “ectomycorrhizal” families (e.g., Fagaceae, Pinaceae, and Betulaceae) but also from other plant species (Fig. 2; Additional file 6: Data S6). In addition, the data included network links between arbuscular mycorrhizal fungi and ectomycorrhizal plant species (Additional file 6: Data S6) as reported previously [64]. When such plant–fungus associations that do not fall into classic categories of mycorrhizal symbioses [63] were excluded from the dataset, network properties changed to some extent (Fig. 3; Additional file 8: Figure S1). Specifically, ectomycorrhizal partial networks displayed lower connectance and nestedenss when non-ectomycorrhizal plant species/taxa were excluded from the data matrices (compare EcM with EcM.ex), while arbuscular mycorrhizal networks remained unchanged after removing ectomycorrhizal and non-mycorrhizal plant species/taxa (compare AM with AM.ex) (Fig. 3; Additional file 8: Figure S1). Meanwhile, although associations between ectomycorrhizal fungi and arbuscular mycorrhizal plants (or arbuscular mycorrhizal fungi and ectomycorrhizal plants) [63] seldom attract attention and they are often removed from high-throughput sequencing datasets before statistical analyses, some of those unusual associations may represent important ecological interactions. An ectomycorrhizal fungus in the truffle genus (*Tuber melanosporum*), for instance, is known to cause severe necrosis in root cortices of non-ectomycorrhizal herbaceous plants [85]. Thus, for the standardization of plant–fungus network analyses inferred with high-throughput sequencing, it is important to emphasize the possibility that network links can represent not only mutualistic but also neutral and antagonistic interactions [17]. Given also that even well-known combinations of plant and fungi can result in antagonistic interactions depending on soil environmental conditions and host plant nutrition [76, 86], potential diversity of ecological interactions within a network and its community-scale consequences [79] deserve intensive research.

Our community-scale comparative analysis targeting a latitudinal range from cool-temperate to subtropical regions has some implications for geographic diversity patterns of plant-associated fungi, although careful interpretation is required given the small number of study sites. The number of detected ectomycorrhizal fungal OTUs was lower in subtropical than in temperate forests (Fig. 3a), presumably reflecting geographic variation in the relative abundance of Fagaceae, Pinaceae, and Betulaceae plants in plant communities as discussed in previous studies [87–90] (see also [91]). In contrast, the number of arbuscular mycorrhizal fungal OTUs increased towards south in our data, while a previous meta-analysis detected no latitudinal diversity gradient regarding the fungal functional group [92] (see also [93]). The total number of fungal OTUs was also higher in subtropical forests, peaked in the southernmost site. Interestingly, unlike other study sites, the entire plant–fungus associations of the southernmost sampling site was characterized by low levels of network-scale interaction specificity and host plant differentiation as well as by the absence of anti-nested network architecture. Although some pioneering studies have investigated host preferences of tropical fungi [94–96], it remains a challenge to examine whether plant–fungus network structures differ substantially between forests in subtropical/tropical regions and those in temperate regions.

## Conclusions

Based on the large datasets of root-associated fungi, we herein showed how plant–fungus network architecture varied across the Japanese Archipelago. For further understanding the diversity of below-ground pant–fungus associations, more comparative studies of community-scale characteristics are required especially in the tropics. Moreover, further data of networks consisting of pathogenic fungi and their host plants are awaited to discuss community-scale properties of negative plant–soil feedbacks [97]. Given that the number of pathogenic fungi included in our present analysis was too few to evaluate statistical features of their networks, selective sampling of pathogen-infected plant individuals may be necessary. Improving reference databases of fungal functions by conducting a series of inoculation experiments is also an important challenge towards better understanding of the roles of fungal communities. In addition, to gain comprehensive understanding of plant–soil feedbacks in terrestrial communities, we need to reveal the structures of networks involving not only fungi but also bacteria and archaea [98]. More macroecological studies of plant–microbe interactions [82, 99, 100], along with experimental studies testing functions of poorly characterized microorganisms [11, 13, 14], will reorganize our knowledge of terrestrial ecosystem processes.

## Additional files

Additional file 1: Data S1. Information of study sites, taxonomic and functional-group information of the fungal OTUs detected. (XLSX 1128 kb)

Additional file 2: Data S2. Sample-level matrices of plant–fungus associations. (XLSX 8386 kb)

Additional file 3: Data S3. Sequences of the non-glomeromycete fungal OTUs detected. (TXT 2166 kb)

Additional file 4: Data S4. Sequences of the glomeromycete fungal OTUs detected. (TXT 320 kb)

Additional file 5: Data S5. Species-level matrices of plant–fungus associations. (XLSX 1237 kb)

Additional file 6: Data S6. Network data matrices. (XLSX 1886 kb)

Additional file 7: Data S7. Results of the randomization analysis. (XLSX 23 kb)

Additional file 8: **Figure S1.** Principal component analysis of network properties. (PDF 158 kb)

## Abbreviations

ANOVA: analysis of variance
DDBJ: DNA Data Bank of Japan
ITS: Internal transcribed spacer
LCA: Lowest common ancestor
OTU: Operational taxonomic unit
QCauto method: Query-centric auto-*k*-nearest neighbor method
