# Supplementary material for: Structural diversity across arbuscular mycorrhizal, ectomycorrhizal, and endophytic plant–fungus networks
Source: BMC Plant Biol. 2018 Nov 21;18:292. doi: 10.1186/s12870-018-1500-5 (PMC6249749; doi:10.1186/s12870-018-1500-5)
Supplement: Supplementary file 8 — Figure S1. Principal component analysis of network properties. (PDF 158 kb) [file 12870_2018_1500_MOESM8_ESM.pdf]

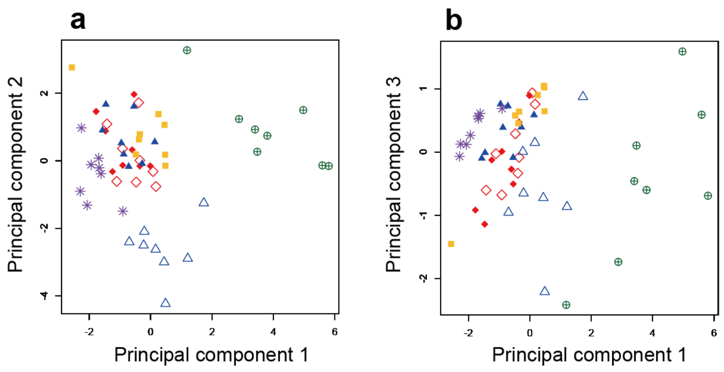

Factor loadings:

Variable	PC1	PC2	PC3
Number of plant species/taxa	-0.013	0.921	-0.201
Number of fungal OTUs	0.818	0.365	-0.359
Fungus/plant ratio	0.811	-0.349	-0.428
Connectance	-0.059	-0.912	-0.296
Relative interaction specificity	0.884	0.236	0.046
Relative nestedness	-0.824	0.274	-0.365
Relative checkerboard score	0.904	-0.044	0.309

Additional File 8: Fig. S1 Principal component analysis of network properties. **a** Principal component 1 vs. principal component 2. Factor loadings of the examined variables are shown on the right. **b** Principal component 1 vs. principal component 3.
